# Primary care utilization and satisfaction among people with cancer in Sweden: a cross-sectional survey study

**DOI:** 10.1186/s12875-026-03623-9

**Published:** 2026-10-26

**Authors:** Lars Garpenhag, Sixten Borg, Linn Rosell, Anna-Maria Larsson, Susanna Calling

**Affiliations:** 1 Division of Psychiatry, Department of Clinical Sciences Lund, Lund University, Lund, Sweden; 2 Center for Primary Health Care Research, Department of Clinical Sciences Malmö, Lund University/Region Skåne, Malmö, Sweden; 3 Regional Cancer Centre South, Lund, Sweden; 4 Department of Health Sciences, Faculty of Medicine, Lund University, Lund, Sweden; 5 Division of Oncology, Department of Clinical Sciences Lund, Lund University, Lund, Sweden; 6 University Clinic Primary Care Skåne, Region Skåne, Malmö, Sweden

**Keywords:** Primary care, Cancer care, Cancer survivors, Healthcare satisfaction, Healthcare organization, Sweden

## Abstract

**Background:**

Primary care services are important to meet the complex needs of people living with or having survived cancer. However, there is a need for improved knowledge about how this patient group use and experience primary care. The aim of this study was to describe the utilization of primary care in people with cancer 1–2 years and 5–6 years after diagnosis, to assess satisfaction with post-diagnosis primary care contacts, and to identify patient and disease characteristics and utilization patterns associated with satisfaction.

**Methods:**

Data from a cross-sectional digital survey were used to measure primary care utilization and satisfaction among people diagnosed with breast, prostate, lung, and colorectal cancer 1–2 and 5–6 years ago. T-tests and chi2-tests were used to detect differences, and logistic regression to identify associations between satisfaction and patient, disease and caregiver characteristics.

**Results:**

Almost all participants had been in contact with primary care since being diagnosed with cancer. A majority had been so for cancer-related reasons, even though most had only been in contact for routine matters. Among participants who had completed cancer treatment, few reported that they had been actively handed over to primary care. Around half of the participants reported that their post-diagnosis primary care contacts had been satisfactory, while 8% rated them as dissatisfactory. However, rates of satisfaction differed considerably between subgroups. Satisfaction was positively associated with having a regular physician and with active handovers, and negatively associated with problem-induced cancer-related primary care contacts and with an absence of cancer-related contacts, as well as with number of comorbidities. Overall, differences based on time since diagnosis were small.

**Conclusions:**

Most people utilize primary care after a cancer diagnosis, including for cancer-related reasons. Many are satisfied with their primary care contacts, but satisfaction varies between sub-groups and is associated with factors related to patients’ health, the nature of contacts, and their relationship to primary care providers.

**Clinical trial number:**

Not applicable.

**Supplementary information:**

The online version contains supplementary material available at https://doi.org/10.1186/s12875-026-03623-9.

## Introduction

Due to rises in the incidence and prevalence of cancer [1], healthcare systems are confronted with the challenge of fulfilling the oftentimes complex needs of a growing number of people living with or having survived the disease. Swedish estimates suggest a national 10-year cancer prevalence of 640 000 in 2040 accompanied by vast increases in societal costs [2]. People with cancer experience diverse health and rehabilitation needs, which may endure or set in long after treatment completion and are not always met [3–5]. Increasing survival emphasize the need to manage cancer as a chronic disease, as part of a wider multimorbidity in aging populations [6–8]. These concerns have put primary care services in the spotlight.

Regular primary care involvement in the care for people with cancer has been proposed to improve the continuity of care [9] and to be a possible avenue to more cost-efficient use of healthcare resources [10]. However, findings are inconclusive regarding preferences on how responsibilities should be divided between primary care and cancer specialists [11–13].

Internationally, studies show that people who have received a cancer diagnosis tend to utilize primary care services more frequently than patients in general [14, 15], and suggest that levels of utilization may be elevated for years [16–18]. Contact reasons that contribute to increased primary care use include management of cancer and cancer treatment related symptoms, informational and psychological needs, and, in some national contexts, routine surveillance [16, 19–21].

Meanwhile, evidence suggest that people with cancer encounter challenges that may limit their access to satisfactory primary care [13, 16, 22–24]. Most research measuring primary care satisfaction in people with cancer has been focused on the evaluation of specific models of follow-up care, or intervention measures in the form of intensive or proactive primary care contacts [25–32]. However, studies mainly focused on cancer survivors in the US have found high levels of satisfaction with primary care providers participations in general post-treatment care [33–35]. A study on Dutch survivors of prostate cancer found a strong positive association between satisfaction with one’s primary care physician and primary care utilization during follow-up [36].

In Sweden, the primary care sector is assigned a key role in ongoing efforts to enhance the delivery of person-centered care [37]. National cancer rehabilitation guidelines state that primary care services should be present throughout the cancer care trajectory, e.g., to provide basic forms of cancer rehabilitation and palliative care. If there is need for it, cancer specialists are to refer patients to primary care by active handover [38]. However, these guidelines are not fully implemented [39]. Qualitative studies have indicated communication problems between cancer specialist and primary care providers and that people with cancer encounter barriers to well-functioning primary care access and may experience their contacts with primary care as challenging [24, 40, 41]. Still, there remains a lack of evidence regarding both primary care utilization patterns and patients’ satisfaction with their primary care contact following a cancer diagnosis.

The aim of this study was (i) to describe the utilization of primary care in people with cancer, 1–2 years and 5–6 years after diagnosis, (ii) to assess their general satisfaction with primary care contacts since the cancer diagnosis, and (iii) to identify patient and disease characteristics and utilization patterns associated with satisfaction. The choice to study this at two distinct points in time after the diagnosis was motivated by a wish to enable comparison between experiences of people recently diagnosed and those who have survived for some time.

## Methods

The study was cross-sectional based on self-reported data collected in an online survey and data from the Swedish cancer registry. The study was approved by the Swedish Ethical Review Authority (file no. 2022-05569-01) and was conducted in accordance with the Declaration of Helsinki. All participants gave their informed consent.

### Setting

The catchment area of the study was the Southern Healthcare Region, Sweden (pop. ~1.9 million), encompassing county council regions Skåne, Blekinge, Kronoberg and southern Halland. In Sweden, primary care services are mainly publicly financed and offered against subsidized fees to citizens and residents, by public as well as private providers. Most primary healthcare centers (PHCC) deliver care by multidisciplinary teams that may include physicians, nurses, and rehabilitation professionals such as physiotherapists and psychologists. As a part of efforts to promote the continuity of care, patients are to be offered an assigned regular primary care physician. However, the actual share of patients assigned a regular physician varies widely between different regions [42].

### Participants

Participants had to fulfill the following inclusion criteria: (i) ≥ 18 years of age, (ii) diagnosed with either invasive breast, prostate, lung, or colorectal cancer (including metastasized cancers) 1–2 years or 5–6 years before study start, and (iii) residence in the catchment area at diagnosis as well as at study enrolment.

### Data collection

Data from the Swedish Cancer Registry was used to identify all individuals who were eligible according to the inclusion criteria, and data were collected on age, sex, diagnosis, date of cancer diagnosis, region, address, and vital status. In May 2023, identified individuals were mailed an invitation to participate in the online survey, together with study information. A reminder was sent after 1 month, and the survey was closed after 2½ months.

Survey data were collected using the electronic data capture application REDCap [43, 44]. Questionnaire access required participants to use a personal code. Consent was collected with a mandatory item.

The questionnaire (please see supplementary file) was constructed specifically for the current research project. Items were constructed based on relevant literature, and in particular the results of a qualitative pre-study [24, 41]. In addition to survey-specific items on demographics and socioeconomic status, cancer and cancer treatment, primary care contacts and needs of next-of-kin, the EORTC QLG Core Questionnaire (EORTC-QLQ-C30) [45], the Assessment of Rehabilitation Needs Checklist (ARNC) [46] and one item from the 36-Item Short Form Health Survey (SF-36) [47] were used to collect data on health-related quality of life (HRQoL), current health needs and self-assessed general health.

### Variables

#### Relationship to healthcare services

*Region of residence*: county council region in which primary care is provided.

*Organization of current PHCC operator*: private, public or unknown.

*Current regular primary care physician*: Whether the participant has been assigned one or not or does not know.

*Cancer-related primary care contacts*: Individual indicators were used for reasons of post-diagnosis contact (blood sampling, assistance with aids (e.g., PICC-line, ostomy, catheter), physical problems, psychological/social problems, other reasons), as well as the total number of reasons stated. In addition, three categories of cancer-related primary care contact patterns were constructed based on the reasons reported:

- *Routine contacts only*: the participant has reported having contacts, but *only* for blood sampling or assistance with aids (tasks that are routinely carried out at PHCCs for patients with cancer, following a referral from cancer care).
- *Any problem-induced contacts*: the participant has reported any combination of reasons that include contacts for physical problems, psychological/social problems, or other reasons associated with cancer.
- *No cancer-related contacts*: the participant has reported that they have not had any post-diagnosis cancer-related contacts.

*Regular primary care contacts to manage other chronic condition(s)*: yes or no (for participants who indicated having comorbidities in a preceding item).

*Contact with primary care since diagnosis*: The proportion of participants who had been in contact with primary care was derived using all available items related to contacts.

*Care pathway position*: factor indicating whether a participant was subject to planned or ongoing cancer treatment, regardless of intention (yes, no, unknown to the participant), and if the participant – if not in treatment – was actively handed over to a PHCC after finished cancer treatment (yes, no, unknown to the participant).

*Travel distance to primary healthcare center*: categorized as 0–29, 30–59 or ≥ 60 min.

#### Satisfaction with primary care contacts

*General satisfaction with primary care contacts following the cancer diagnosis* was measured with one item asking participants to rate how well contacts had worked on a five-point scale, from “very well” to “very poorly”. The item also permitted participants to respond that they did not know/had not been in contact with primary care.

For descriptive tabulation, we categorized patients’ satisfaction with primary care contacts into satisfied (“very well” or “well”), neutral (“neither well nor poorly”), dissatisfied (“poorly” or “very poorly”), and uncertain (“do not know/no contacts”). In an analysis of factors explaining satisfaction, we dichotomized responses as satisfied (“very well” or “well”), and neutral/dissatisfied (“very poorly”, “poorly” or “neither well nor poorly”).

#### Patient background characteristics

*Age*: subdivided into age groups.

*Sex*: men and women.

*Diagnosis*: invasive breast, lung, prostate, or colorectal cancer.

*Time since diagnosis*: 1–2 or 5–6 years before study start.

*Native language*: Swedish or non-Swedish.

*Level of education*: categorized as at most senior high school or post-high school.

#### Well-being

*Indicators of individual comorbidities*: self-reported high blood pressure, cardiovascular disease, diabetes, chronic obstructive pulmonary disease, asthma, thyroid disease, dyspepsia, irritable bowel disease, pain, anxiety/depression, or other chronic illness.

*Number of comorbidities*: the number of individual comorbidities reported (tabulated categorized as 0, 1, 2, 3 or more).

*Number of current cancer-related health problems*: Number of problems in the last month reported in the ARNC, an instrument asking the respondent to assess to what degree (No problems/Minor problems/Major problems/Very major problems) their cancer have affected 19 health-related areas, including, e.g., sleep, pain and physical activity (we used the 19-item rather than the 21-item version of the checklist) [46].

*Health Related Quality of life*: to assess HRQoL we used the one-item measure general health from the SF-36: “In general, would you say your health is: Excellent/Very good/Good, Fair/Poor)” [47], as well as the functioning scales Physical function, Role function, Emotional function, Cognitive function and Social function, and symptom scales Fatigue, Pain, Dyspnea, Sleep, Appetite loss, Nausea and vomiting, Financial issues, Diarrhea, Constipation and Financial difficulties, from the EORTC-QLQ-C30 [45,48].

*Current sick-leave, overall and cancer-related, respectively*: yes or no.

### Statistical analysis

To identify factors associated with satisfaction with primary care we employed descriptive statistics and a logistic regression model. Significance testing was used to detect associations, t-tests of continuous variables and chi-square test of categorical variables. P-values < 0.05 were considered statistically significant and were used as a flagging device of differences of interest for hypothesis formulation and further investigation. We made no adjustment för multiple testing due to the exploratory nature of the analyses, and p-values should therefore be interpreted with caution.

In the logistic regression model, we investigated the variables listed above concerning the categories patient characteristics, relation to healthcare, and well-being. Some variables were available in different levels of aggregation, e.g. total number of comorbidities versus individual indicators of each of eleven comorbidities, general health versus individual functional scales, etc. All variables within each category were tested, one aggregation level at a time. To decide whether the investigated variables provided explanation of satisfaction and kept to be included in the final model, testing was carried out using a cross-validation approach. In each step we reviewed the model as follows: (a) The sample was split in half, and the model was fit separately to both halves. A variable was accepted as providing explanation if it became significant (*p* < 0.05) in both halves. (b) The model was fitted to two thirds of the sample, and its prediction characteristics were evaluated in the remaining third of the sample. These characteristics were accuracy (fraction correctly predicted) and area under the curve (AUC). There are several rules of thumb for judging AUC. We adopted the rules proposed by Hilbe [49], interpreting 0.5–0.65 as having little predictive power; 0.65–0.80 as moderate; and above 0.80 as strong. The threshold for discriminating between predicted positive and negative cases was taken as the level maximizing Youden’s J statistic [49]. By default, the model used satisfaction dichotomized as described above. A sensitivity analysis examined its stability with regards to cut-off level for satisfaction.

## Results

### Sample and participant characteristics

Invited and responding participants are described in Table 1. In total, 2131 individuals responded to the survey, out of which 1278 had been diagnosed with cancer 1–2 years before and 853 5–6 years before. The overall response rate was 26%. People ≥ 80 years old and people with lung cancer displayed substantially lower response rates (12% and 17% respectively), while the rates were substantially higher for people in ages 18–69 years (33–36%). 56% of participants were men. People 70–79 years formed the largest age group (38%) and people 18–49 years the smallest (7%). The majority, 60%, had received their diagnoses 1–2 years before study start.

**Table 1:** Participant background characteristics and response rates

	Participants	Invited	Response rate
	*n* (%)	*N*	%
**All**	2131 (100)	8262	26
**Gender**			
Men	1196 (56)	4 510	27
Women	935 (44)	3 752	25
**Age (years)**			
18–49	146 (7)	434	34
50–59	327 (15)	922	35
60–69	644 (30)	1956	33
70–79	817 (38)	3269	25
80–	197 (9)	1681	12
**Diagnosis**			
Breast cancer	720 (34)	2 639	27
Prostate cancer	906 (43)	3 317	27
Lung cancer	100 (5)	603	17
Colorectal cancer	405 (19)	1 703	24
**Time since diagnosis**			
1–2 years	1278 (60)	4 763	27
5–6 years	853 (40)	3 499	24
Level of education *			
Post-high school	1101 (52)		
Native language *			
Swedish	1997 (94)		
Other language	134 (6)		

Notes: * As data on educational level and native language were obtained from the questionnaire, the response rates cannot be estimated

### Primary care utilization

According to the self-reported data, almost all participants (98%) had been in contact with primary care at least once since the cancer diagnosis, and 78% had been in contact due to reasons related to cancer or cancer treatment (Table 2). The most common cancer-related reason for contact was blood sampling (72%), followed by assistance with aids (19%). 59% had exclusively had such routine character contacts, leaving 19% in total (18% in the group diagnosed 5–6 years ago) who had been in contact because of physical problems (12%), psychological/social problems (5%) or other cancer-related reasons (7%). 41% were in regular contact with primary care for the management of non-cancer chronic disease. 47% reported having a regular primary care physician. Among participants who were neither undergoing cancer treatment nor planned for treatment, 15% were aware of having been actively handed over to primary care at treatment conclusion.

Compared to participants diagnosed 5–6 years ago, a greater share of participants diagnosed 1–2 years ago reported experience of cancer-related primary care contacts (80% vs. 74%) and cancer-related blood sampling at the PHCC (74% vs. 68%).

**Table 2:** Participants’ primary care contacts since the cancer diagnosis

	1–2 years	5–6 years	All
	*n* (%)	*n* (%)	*n* (%)
**Total number of participants**	1278 (100)	853 (100)	2131 (100)
**Post-diagnosis primary care contact**	1254 (98)	833 (98)	2087 (98)
**Cancer-related reasons for contact**	1026 (80)	632 (74)	1658 (78)
Routine contacts			
Blood sampling	950 (74)	583 (68)	1533 (72)
Assistance w. Aids	246 (19)	149 (17)	395 (19)
Problem-induced contacts			
Due to physical problems	153 (12)	98 (11)	251 (12)
Due to psychological or social problems	65 (5)	47 (6)	112 (5)
Due to other reasons	90 (7)	50 (6)	140 (7)
**Patterns of cancer-related contacts**			
Routine contacts only	777 (61)	482 (57)	1259 (59)
Any problem-induced contacts	249 (19)	150 (18)	399 (19)
No cancer-related contacts	252 (20)	221 (26)	473 (22)
**Regular contacts to manage other chronic condition(s)**	519 (41)	344 (40)	863 (40)
**PHC operator**			
Public PHCC	739 (58)	492 (58)	1231 (58)
Private PHCC	477 (37)	320 (38)	797 (37)
Do not know	62 (5)	41 (5)	103 (5)
**Current regular primary care physician**			
Yes	581 (45)	409 (48)	990 (46)
No	588 (46)	365 (43)	953 (45)
Do not know	109 (9)	79 (9)	188 (9)
Active handover after cancer treatment*			
Yes	151 (16)	107 (14)	258 (15)
No	489 (51)	405 (54)	894 (52)
Do not know	319 (33)	243 (32)	562 (33)

Notes: * The statistics on active handovers only include participants neither currently in cancer treatment nor planned for treatment (1 714 participants in total)

### Patterns of cancer-related primary care contacts

The participants with *no cancer-related contacts* were predominantly female (66%) and diagnosed with breast cancer (55%) (Table 3). A similar pattern was seen for the group who reported *any problem-induced contacts* (61% women, 49% breast cancer). However, among participants who reported *routine contacts only*, the majority were men (70%), and the most common cancer diagnosis was prostate cancer (55%).

**Table 3:** Participant characteristics by cancer-related primary care contact pattern

	No cancer-related contacts	Routine contacts only	Any problem-induced contacts	Total	P*
	*n* (%)	*n* (%)	*n* (%)	*n* (%)	
**All**	473 (100)	1259 (100)	399 (100)	2131 (100)	
**Gender**					< 0.0001
Women	313 (66)	378 (30)	244 (61)	935 (44)	
Men	160 (34)	881 (70)	155 (39)	1196 (56)	
**Age (years)**					< 0.0001
18–49	25 (5)	56 (4)	65 (16)	146 (7)	
50–59	67 (14)	167 (13)	93 (23)	327 (15)	
60–69	149 (32)	401 (32)	94 (24)	644 (30)	
70–79	166 (35)	531 (42)	120 (30)	817 (38)	
80-	66 (14)	104 (8)	27 (7)	197 (9)	
**Diagnosis**					< 0.0001
Breast	261 (55)	264 (21)	195 (49)	720 (34)	
Lung	22 (5)	57 (5)	21 (5)	100 (5)	
Prostate	104 (22)	698 (55)	104 (26)	906 (43)	
Colorectal	86 (18)	240 (19)	79 (20)	405 (19)	
**Time since diagnosis**					= 0.0033
1–2 years	252 (53)	777 (62)	249 (62)	1278 (60)	
5–6 years	221 (47)	482 (38)	150 (38)	853 (40)	
Actively handed over**					< 0.0001
Yes	36 (9)	167 (16)	55 (19)	258 (15)	
No	245 (60)	500 (49)	149 (51)	894 (52)	
Do not know	129 (31)	347 (34)	86 (30)	562 (33)	
**No of additional comorbidities**					< 0.0001
0	150 (32)	436 (35)	119 (30)	705 (33)	
1	161 (34)	446 (35)	119 (30)	726 (34)	
2	87 (18)	231 (18)	73 (18)	391 (18)	
3+	75 (16)	146 (12)	88 (22)	309 (15)	
**General health**					< 0.0001
Excellent	45 (10)	126 (10)	22 (6)	193 (9)	
Very good	149 (32)	381 (30)	84 (21)	614 (29)	
Good	185 (39)	516 (41)	159 (40)	860 (40)	
Fair	87 (18)	206 (16)	111 (28)	404 (19)	
Poor	7 (1)	30 (2)	23 (6)	60 (3)	

Notes: * Tests to detect association between indicated factor and contact pattern. ** The statistics on active handovers only include participants neither currently in cancer treatment nor planned for treatment (1 714 participants in total)

Regarding general health, it was most common among participants with *routine contacts only* or *no cancer-related contacts* to rate their health as either good or very good (71% in total in both groups), while in the group who reported *any problem-induced contacts* most rated their health as either good or fair (68% in total).

Among participants with no current/planned cancer treatment, 19% of those with *problem-induced contacts* and 16% of those with *routine contacts only* were aware that they had been actively handed over to primary care. However, among participants with *no cancer related contacts* only 9% knew that they had been actively handed over.

Participants diagnosed with cancer 5–6 years ago were over-represented in the *no cancer-related contacts* group in relation to their proportion of the full sample (47% vs. 40%). While most participants in all contact pattern groups had 0–1 comorbidity, it was significantly more common among those with *no cancer-related contacts* and those with *any problem-induced contacts* to report 3 or more comorbidities (16 and 22% vs. 12%; *p* < 0.0001). The mean (95% CI) number of comorbidities in these contact pattern groups were 1.25 (1.14–1.36), 1.44 (1.31–1.57), and 1.13 (1.07–1.19) among *routine contacts only*. Due to the age structure of the sample, people 60–69 and 70–79 years comprised the largest age groups in all the patterns. However, participants under 60 years made up a greater proportion of those with *any problem-induced contact*s (39% vs. 19% and 17%; *p* < 0.0001). Mean age (95% CI) by contact pattern group was 63.3 (62.1–64.5) among *any problem-induced contacts*, 68.6 (67.7–69.5) among *no cancer-related contacts*, and 68.4 (67.9–68.9) among *routine contacts only*.

### Satisfaction with primary care contacts

48% of participants reported being satisfied with their primary care contacts since the cancer diagnosis and 19% gave a neutral response (see Table 4). A minority, 8%, indicated that they were dissatisfied. In general, differences between sub-groups were small. However, compared to the full sample, a greater proportion of participants aged 18–49 reported being satisfied (54% vs. 48%). People diagnosed with lung cancer displayed both the smallest share of satisfied participants (39%) and the greatest share of dissatisfied participants (17%).

**Table 4:** Satisfaction with primary care contacts since the cancer diagnosis

	Satisfied	Neutral	Dissatisfied	Uncertain	Total
	*n* (%)	*n* (%)	*n* (%)	*n* (%)	*n* (%)
**All**	1014 (48)	401 (19)	181 (8)	535 (25)	2131 (100)
**Gender**					
Women	433 (46)	183 (20)	93 (10)	226 (24)	935 (100)
Men	581 (49)	218 (18)	88 (7)	309 (26)	1196 (100)
**Age**					
18–49	79 (54)	27 (18)	15 (10)	25 (17)	146 (100)
50–59	152 (46)	65 (20)	32 (10)	78 (24)	327 (100)
60–69	292 (45)	116 (18)	53 (8)	183 (28)	644 (100)
70–79	396 (48)	152 (19)	67 (8)	202 (25)	817 (100)
80-	95 (48)	41 (21)	14 (7)	47 (24)	197 (100)
**Diagnosis**					
Breast	336 (47)	138 (19)	74 (10)	172 (24)	720 (100)
Lung	39 (39)	20 (20)	17 (17)	24 (24)	100 (100)
Prostate	429 (47)	168 (19)	62 (7)	247 (27)	906 (100)
Colorectal	210 (52)	75 (19)	28 (7)	92 (23)	405 (100)
**Time since diagnosis**					
1–2 years	630 (49)	235 (18)	100 (8)	313 (24)	1278 (100)
5–6 years	384 (45)	166 (19)	81 (9)	222 (26)	853 (100)
Actively handed over *					
Yes	192 (74)	35 (14)	13 (5)	18 (7)	258 (100)
No	323 (36)	186 (21)	109 (12)	276 (31)	894 (100)
Do not know	267 (48)	99 (18)	32 (6)	164 (29)	562 (100)
**Cancer-related primary care contact pattern**					
Routine contacts only	667 (53)	225 (18)	83 (7)	284 (23)	1259 (100)
Any problem-induced contacts	212 (53)	103 (26)	55 (14)	29 (7)	399 (100)
No cancer-related contacts	135 (29)	73 (15)	43 (9)	222 (47)	473 (100)
**No of additional comorbidities**					
0	345 (49)	112 (16)	43 (6)	205 (29)	705 (100)
1	362 (50)	123 (17)	47 (6)	194 (27)	726 (100)
2	177 (45)	98 (25)	38 (10)	78 (20)	391 (100)
3+	130 (42)	68 (22)	53 (17)	58 (19)	309 (100)
**General health**					
Excellent	103 (53)	19 (10)	11 (6)	60 (31)	193 (100)
Very good	314 (51)	93 (15)	34 (6)	173 (28)	614 (100)
Good	403 (47)	178 (21)	68 (8)	211 (25)	860 (100)
Fair	170 (42)	98 (24)	55 (14)	81 (20)	404 (100)
Poor	24 (40)	13 (22)	13 (22)	10 (17)	60 (100)

* The statistics on active handovers only include participants neither currently in cancer treatment nor planned for treatment (1 714 participants in total)

Among participants who were not in current or planned treatment, those who were aware of having been actively handed over to primary care were satisfied substantially more often (74%) than either those participants who were sure that they had not been so or did not know (36% and 48% respectively), and less often dissatisfied (5% vs. 12% and 6% respectively).

Compared to the full sample, a substantially smaller proportion of participants with no cancer-related primary care contacts since the cancer diagnosis reported being satisfied (29% vs. 48%). Participants with a problem-induced contact pattern, meanwhile, showed greater tendencies to be neutral (26% vs. 19%) and dissatisfied (14% vs. 8%).

Participants with 3 or more comorbidities reported being satisfied less often than the full sample (42% vs. 48%) and were dissatisfied more often (17% vs. 8%). In regard to general health, the share of satisfied participants was the highest among those who assessed their health as excellent (53%) and consistently dropped with decreased levels of reported health to 40%, among those assessing their health as poor. The reverse overall tendency is seen among those reporting dissatisfaction, with participants with poor health having the greatest proportion of dissatisfied (22%). 

### Logistic regression model of satisfaction

In the logistic regression model, experience of active handover and having an assigned regular physician were positively associated with satisfaction. Meanwhile, the following variables were negatively associated with satisfaction: number of additional comorbidities; no active handover; and contact pattern with experience of problem-induced contacts or who had no cancer-related contacts (Table 5). The other investigated variables provided no statistically relevant explanation of satisfaction.

The model correctly predicted the level of satisfaction in 67% of the cases in the test set. The AUC was 0.68, indicating moderate predictive ability.

In the sensitivity analysis using alternative cut-off levels between satisfied and dissatisfied, the contact pattern ceased to be statistically significant because the factor lost its discriminatory ability due to diminishing margins. The other explanatory variables remained significant in the sensitivity analysis.

**Table 5:** Association between primary care satisfaction and explanatory variables: odds ratio (OR) and 95% confidence intervals (CI)

Variables	OR		95% CI
Number of additional comorbidities (cumulative)	0.77	***	0.70–0.84
Care pathway position			
Ongoing/Planned cancer treatment (ref.)	1.00		-
Actively handed over: Yes	1.76	**	1.18–2.63
Actively handed over: No	0.52	***	0.39–0.69
Actively handed over: Don’t know	0.90	ns	0.65–1.24
Cancer-related primary care contact pattern			
Routine contacts only (ref.)	1.00		-
Any problem-induced contacts	0.66	**	0.51–0.85
No cancer-related contacts	0.60	***	0.45–0.81
Has a regular primary care physician (ref. = has not)	1.88	***	1.50–2.35

Notes: Ref = the factor’s reference level in the model. OR < 1 means less likelihood of satisfaction; OR > 1 means greater. ns = not significant. * *p* < 0.05, ** *p* < 0.01, *** *p* < 0.001

## Discussion

This study is the first, to our knowledge, to report quantitatively on the utilization of, and satisfaction with, primary care among people with cancer in Sweden. Almost all participants had been in contact with primary care since being diagnosed with cancer, and many had been so for cancer-related reasons. In most cases, however, cancer-related contacts had been limited to the handling of routine matters (e.g., blood sampling), while experiences of contacts concerning physical problems, psychological/social problems and other reasons related to cancer were considerably less common. The proportion of participants who reported having been in contact with primary care because of cancer-related problems seems small in relation to, e.g., a study on Dutch colon cancer survivors, which found cancer-related symptoms to be a common reason for primary care contacts [21]. It is worth noting that while the patterns described in our study tell us about the role played by primary care, it may not necessarily be reflective of either participants’ healthcare needs or the fulfillment of these needs, since such problems might have been handled through other healthcare contacts, e.g., with specialized cancer care. In the Swedish health care system, cancer specialist clinics may offer follow-up for many years after diagnosis, hence patients may turn to the hospital with questions regarding issues connected to previous or current cancer disease, which might explain the low percentage contacting primary care with these issues.

Despite that two county regions in our catchment area have among the lowest percentages of patients officially assigned a regular primary care physician [42], the share of participants aware of having a regular primary care physician was high compared with that of the Swedish general population (46% vs. 32%), fitting a general pattern of higher rates among people with more serious health needs [50]. However, few of the participants who had finished cancer treatment reported that they had been subject to an active handover. While many did not know if they had undergone one or not, the success of an active handover that remains unknown to the patient is questionable. The low rates of confirmed active handovers mirrors findings in a recent report mapping cancer rehabilitation in Sweden, emphasizing challenges with coordination between cancer and primary care providers, most prominently post-treatment, resulting in unequal access to cancer rehabilitation [39]. Similarities in primary care utilization observed among participants diagnosed 1–2 and 5–6 years ago respectively suggest a stability in the post-diagnosis relationship to primary care.

Almost half of the participants were satisfied with their post-diagnosis primary care contacts, while only 8% were dissatisfied. In the absence of directly comparable data, we cannot say for certain how this compares to experiences in the general population. However, a survey found that 78% of Swedish patients perceived primary care appointments to be positive regarding participation, co-creation of care, reception, and communication [51]. While our measure of satisfaction was broader and more open, prohibiting a direct comparison, the lower satisfaction rate found in our study could suggest that people with cancer are less satisfied than Swedish primary care patients in general.

Satisfaction rates were largely similar regardless of time since diagnosis but differed considerably between subgroups according to a number of other criteria. This emphasizes the need to highlight circumstances impacting on satisfaction and thus relevant for primary care providers. In logistic regression, participants’ number of comorbidities was found to be negatively associated with primary care satisfaction. This could be an effect of the treatment burden associated with multimorbidity, i.e., the amount of work and energy a patient must invest in order to adhere to treatments and manage healthcare contacts [52]. In addition, although self-reported health status did not make it into our final model, the observation that satisfaction was lower among patients with lower levels of self-reported general health is in accordance with previous research that has found negative associations between health status and patient satisfaction [53].

Significant negative associations were also found between primary care satisfaction and the experience of problem-induced cancer-related primary care contacts, as well as with an absence of cancer-related contacts. The negative association between satisfaction and problem-induced cancer-related contacts could be explained by insufficient knowledge about cancer and cancer treatment among primary care professionals, which would make it more difficult to correctly diagnose and treat cancer-related problems. This is an issue highlighted in several studies [54], and research on cancer survivors’ satisfaction with primary care in the post-treatment period have found weak endorsement of providers’ abilities related to cancer-specific dimensions of post-treatment care [33–35]. The negative association between satisfaction and a pattern of no cancer-related contacts, meanwhile, might be the result of participants with poor prior experiences refraining from seeking cancer-related care at the PHCC, of participants failing to access primary care to manage cancer-related problems experiencing services as poor as a result, or a combination of these. As the contact pattern groups differed in their composition regarding demographics and cancer diagnoses, some groups would be more affected than others. Within both the group with no cancer-related contacts and the group with problem-induced contacts, women were in the majority and breast cancer was the most common diagnosis.

We found positive associations between the level of satisfaction with primary care and having a regular physician and, among participants not in cancer treatment, with the experience of an active handover to primary care. Personal continuity in healthcare is known to have numerous beneficial effects, including enhanced patient satisfaction [55, 56]. Similarly, active handovers are considered important to ensure care continuity and secure information transfer between different caregivers [38].

### Implications

Knowledge of how people with cancer utilize and experience primary care is crucial for efforts both to integrate primary care services in the cancer care process and to enhance their quality. Our results clearly indicate that people with cancer have a presence at the PHCCs, not least to manage routine care associated with the cancer disease. While routine care seems to work relatively well, the negative associations between primary care satisfaction and the utilization of primary care to deal with cancer-related problems – as well as with non-utilization – suggest that not all needs are currently being met in the group. This calls for training or awareness-raising measures to improve primary care providers’ readiness to deal with consequences of cancer, but also for measures to enhance communication between cancer care and primary care specialists, and thus the continuity of care. In regard to the latter, routinized use of active handovers and survivorship care plans [57] could both prove beneficial. The introduction of such routines, however, is not without challenges, and their implementation requires time as well as resources and organizational support to professionals in both cancer and primary care [23]. The cumulative negative association between primary care satisfaction and comorbidities, meanwhile, tell us that people with multimorbidity may require extra attention in regard to the coordination of care following a cancer diagnosis. Finally, both variables that were found to be positively associated with satisfaction – the provision of regular primary care physicians and active handovers at the conclusion of cancer treatment – are dependent on the organization of healthcare, and thus malleable by policy. The results should serve as an encouragement to decision-makers to promote them.

### Strengths and limitations

A strength of the current study is that we combined our questionnaire study with cancer registry data, deterministically linked on the individual level, thus obtaining both the patients’ views on e.g. satisfaction and clinical data from a high-quality source. The fair size of the study sample is another strength.

The study was observational, and thus we cannot draw conclusions regarding causality. That we relied on self-reported data from patients could be considered a further limitation, which introduces a risk of recall bias. Not all participants, especially in the group diagnosed 5–6 years ago, may correctly recall what kinds of healthcare contacts they have had, or their experience of how well these contacts have worked. However, we believe that the risk for recall bias is mitigated somewhat by the fact that we do not measure frequency of contacts, or discrete dimensions of satisfaction.

Although the response rate was acceptable, there is a risk for non-response bias. The tendency to respond could be greater in, e.g., individuals who have strong feelings about the subject (leading to more polarized data) [58], or who are healthier. This could have influenced our results. We also know that response rates varied based on cancer diagnosis and age, and was considerably lower for people with lung cancer (17% vs. 24–27% for other cancers) and those 80 years and older (12% vs. 25–35% for other age groups). Thus, interpretations concerning the primary care utilization and satisfaction with contacts in these groups especially should be made with certain caution. In addition, given the relatively poor satisfaction among participants diagnosed with lung cancer especially, the low response rate in this group may also have affected the measurement of satisfaction in the sample as a whole.

A final limitation is the unidimensional approach to the measurement of satisfaction with primary care contacts. The use of a multidimensional measure (e.g., based on a number of questionnaire items measuring different dimensions) could have yielded a richer and more nuanced picture of primary care experiences in the studied sample. In addition, due to the fact that the unidimensional approach left considerable room for interpretation on the part of the respondents, there is a risk that it might have left dimensions that could be considered integral to satisfaction without consideration.

## Conclusions

Most people utilize primary care after receiving a cancer diagnosis, including for reasons related to the cancer disease. Looking to cancer-related contacts specifically, a majority have routine contacts only (blood sampling and assistance with aids) whereas only a minority have contacts to handle other cancer-associated needs. Overall, a majority are either satisfied or feel neutral towards with their primary care contacts. Both utilization and satisfaction patterns were remarkably similar irrespective of time since diagnosis. However, satisfaction varied considerably between sub-groups, and were shown to be associated with factors related to patients’ health, the nature of contacts, and their relationship to primary care providers.

## Supplementary information

Below is the link to the electronic supplementary material.

Supplementary Material 1

## Data Availability

The data that support the findings of this study are restricted by the Swedish Ethical Review Authority in order to protect participants’ privacy.

## Abbreviations

ARNC: Assessment of Rehabilitation Needs Checklist
AUC: Area under the curve
EORTC-QLQ-C30: The European Organisation for Research and Treatment of Cancer Quality of Life Group Core Questionnaire
PHCC: Primary healthcare center
HRQoL: Health-related quality of life
SF-36: The 36-Item Short Form Health Survey

## References

[1] Sung H, Ferlay J, Siegel RL, et al. Global cancer statistics 2020: GLOBOCAN estimates of incidence and mortality worldwide for 36 cancers in 185 countries. CA Cancer J Clin. 2021;71(3):209–49. 10.3322/caac.21660. 33538338

[2] Cancerfonden. Cancerfondsrapporten 2016. [The Swedish Cancer Society Report 2016]. Stockholm: Cancerfonden; 2016.

[3] Stein KD, Syrjala KL, Andrykowski MA. Physical and psychological long-term and late effects of cancer. Cancer. 2008;112(11 Suppl):2577–92. 10.1002/cncr.23448. PMC7047657 18428205

[4] Veloso AG, Sperling C, Holm LV, et al. Unmet needs in cancer rehabilitation during the early cancer trajectory – a nationwide patient survey. Acta Oncol. 2013;52(2):372–81. 10.3109/0284186X.2012.745648. 23320774

[5] Fitch M, Zomer S, Lockwood G, et al. Experiences of adult cancer survivors in transitions. Support Care Cancer. 2019;27:2977–86. 10.1007/s00520-018-4605-3. PMC6597588 30588549

[6] Earle CC, Neville BA. Under use of necessary care among cancer survivors. Cancer. 2004;101(8):1712–9. 10.1002/cncr.20560. 15386307

[7] Blane DN, Lewandowska M. Living with cancer and multimorbidity: The role of primary care. Curr Opin Support Palliat Care. 2019;13:213–9. 10.1097/SPC.0000000000000454. 31348011

[8] Pituskin E. Cancer as a new chronic disease: Oncology nursing in the 21st Century. Can Oncol Nurs J. 2022;32(1):87–92. PMC8849169 35280062

[9] Cooper JM, Loeb SJ, Smith CA. The primary care nurse practitioner and cancer survivorship care. J Am Acad Nurse Pract. 2010;22:394–402. 10.1111/j.1745-7599.2010.00528.x. 20670264

[10] Chan RJ, Crawford-Williams F, Chrichton M, et al. Effectiveness and implementation of models of cancer survivorship care: an overview of systematic reviews. J Cancer Surviv. 2023;17:197–221. 10.1007/s11764-021-01128-1. PMC8594645 34786652

[11] Wallner LP, Li Y, Furgal AKC, et al. Patient preferences for primary care provider roles in breast cancer survivorship care. J Clin Oncol. 2017;35:2942–8. 10.1200/JCO.2017.73.1307. PMC5575964 28700276

[12] Radhakrishnan A, McLeod MC, Hamilton AS, et al. Preferences for physician roles in follow-up care during survivorship: Do patients, primary care providers and oncologists agree? J Gen Intern Med. 2018;34(2):184–6. 10.1007/s11606-018-4690-5. PMC6374274 30284174

[13] Taylor S, Johnson H, Peat S, et al. Exploring the experiences of patients, general practitioners and oncologists of prostate cancer follow-up: A qualitative interview study. Eur J Oncol Nurs. 2020;48:101820. 10.1016/j.ejon.2020.10182. PMC7395829 32932010

[14] Adam R, Watson E. The role of primary care in supporting patients living with and beyond cancer. Curr Opin Support Palliat Care. 2018;12:261–7. 10.1097/SPC.0000000000000369. 30074923

[15] Costa AR, Alves L, Nuno L. Healthcare services and medication use among cancer survivors and their partners: A cross-sectional analysis of 16 European countries. J Cancer Surviv. 2020;14:720–30. 10.1007/s11764-020-00886-8. 32594450

[16] Khan NF, Watson E, Rose PW. Primary care consultation behaviours of long-term, adult survivors of cancer in the UK. Br J Gen Pract. 2011;61(584):197–9. 10.3399/bjgp11X561195. PMC3047313 21375904

[17] Heins M, Schellevis F, Rijken M, et al. Determinants of increased primary health care use in cancer survivors. J Clin Oncol. 2012;30:4155–60. 10.1200/JCO.2012.41.9101. 23071230

[18] Brandenbarg D, Roorda C, Groenhof F, et al. Primary healthcare use during follow-up after curative treatment for colorectal cancer. Eur J Cancer Care. 2017;26:e12581. 10.1111/ecc.12581. 27726218

[19] Heins MJ, Korevaar JC, Rijken PM, et al. For which health problems do cancer survivors visit their General Practitioner? Eur J Cancer. 2013;49(1):211–8. 10.1016/j.ejca.2012.07.011. 22897842

[20] Kendell C, Decker KM, Groome PA, et al. Use of physician services during the survivorship phase: A multi-province study of women diagnosed with breast cancer. Curr Oncol. 2017;24(2):81–9. 10.3747/co.24.3454. PMC5407870 28490921

[21] Duineveld LAM, Molthof H, Wieldraaijer T, et al. General practitioners’ involvement during survivorship care of colon cancer in the Netherlands: Primary health care utilization during survivorship care of colon cancer, a prospective multicentre cohort study. Fam Pract. 2019;36(6):765–70. 10.1093/fampra/cmz028. PMC7006992 31204434

[22] Lundstrøm LH, Johnsen AT, Ross L, et al. Cross-sectorial cooperation and supportive care in general practice: Cancer patients’ experiences. Fam Pract. 2011;28:532–40. 10.1093/fampra/cmr011. 21427204

[23] Love M, Debay M, Hudley AC, et al. Cancer survivors, oncology, and primary care perspectives on survivorship care: An integrative review. J Prim Care Community Health. 2022;13:1–9. 10.1177/21501319221105248. PMC9189519 35678264

[24] Garpenhag L, Halling A, Larsson AM, et al. The role of primary care in the cancer care continuum: A qualitative study of cancer survivors’ experiences. Scand J Prim Health Care. 2023;41(1):13–22. 10.1080/02813432.2022.2145848. PMC10088974 36380504

[25] Grunfeld E, Fitzpatrick R, Mant D, et al. Comparison of breast cancer patient satisfaction with follow-up in primary care versus specialist care: results from a randomized controlled trial. Br J Gen Pract. 1999;49:705–10. PMC1313497 10756611

[26] Murchie P, Nicolson MC, Hannaford PC, et al. Patient satisfaction with GP-led melanoma follow-up: A randomized controlled trial. Br J Cancer. 2010;102:1447–55. 10.1038/sj.bjc.6605638. PMC2869159 20461089

[27] Bergholdt SH, Søndergaard J, Larsen PV, et al. A randomised controlled trial to improve general practitioners’ services in cancer rehabilitation: Effects on general practitioners’ proactivity and on patients’ participation in rehabilitation activities. Acta Oncol. 2013;52:400–9. 10.3109/0284186X.2012.741711. 23173759

[28] Holtedahl K, Norum J, Anvik T, et al. Do cancer patients benefit from short-term contact with a general practitioner following cancer treatment? A randomised, controlled study. Support Care Cancer. 2005;13:949–56. 10.1007/s00520-005-0869-5. 16025260

[29] Wattchow DA, Weller DP, Esterman A, et al. General practice vs surgical-based follow-up for patients with colon cancer: Randomised controlled trial. Br J Cancer. 2006;94:1116–21. 10.1038/sj.bjc.6603052. PMC2361245 16622437

[30] Emery JD, Jefford M, King M, et al. ProCare Trial: A phase II randomized controlled trial of shared care for follow-up of men with prostate cancer. BJU Int. 2017;119:381–9. 10.1111/bju.13593. 27431584

[31] Gaillard V, Tricard T, Rebel S, et al. Primary care–based follow–up for prostate and kidney cancer survivors: A retrospective monocentric study. Support Care Cancer. 2022;30:7293–302. 10.1007/s00520-022-07151-x. 35604498

[32] Perfors IAA, Helsper CW, Noteboom EA, et al. Effects of structured involvement of the primary care team versus standard care after a cancer diagnosis on patient satisfaction and healthcare use: the GRIP randomized controlled trial. BMC Prim Care. 2022;23:145. 10.1186/s12875-022-01746-3. PMC9166421 35659264

[33] Mao JJ, Bowman MA, Stricker CT, et al. Delivery of Survivorship Care by Primary Care Physicians: The Perspective of Breast Cancer Patients. J Clin Oncol. 2009;27:933–8. 10.1200/JCO.2008.18.0679. 19139437

[34] Christian A, Hudson SV, Miller SM, et al. Perceptions of primary care among breast cancer survivors: The effects of weight status. Health Serv Res Manag Epidemiol. 2015;2:2333392815587487. 10.1177/2333392815587487. PMC4480862 26120589

[35] Nyarko E, Metz JM, Nguyen GT, et al. Cancer survivors’ perspectives on delivery of survivorship care by primary care physicians: an internet-based survey. BMC Fam Pract. 2015;16:143. 10.1186/s12875-015-0367-x. PMC4615334 26486311

[36] Wollersheim BM, Eenbergen MV, Asselt KMV, et al. Type of treatment, symptoms and patient satisfaction play an important role in primary care contact during prostate cancer follow-up: Results from the population-based PROFILES registry. BMC Fam Pract. 2021;22:218. 10.1186/s12875-021-01567-w. PMC8569955 34736413

[37] Janlöv N, Blume S, Glenngård AH, et al. Sweden: Health system review. Health Syst Transition. 2023;15(4):1–236. 38230685

[38] Regionala cancercentrum i samverkan. Cancerrehabilitering: Nationellt vårdprogram [Cancer rehabilitation: National care program]. 4th version. In: Kunskapsbanken. Regionala cancercentrum i samverkan. 2023. https://kunskapsbanken.cancercentrum.se/globalassets/vara-uppdrag/rehabilitering-palliativ-vard/vardprogram/nationellt-vardprogram-cancerrehabilitering.pdf. Accessed 20 Oct 2024. Swedish.

[39] Socialstyrelsen. Fortsatt utveckling av cancerrehabiliteringen: Kartläggning av regionernas cancerrehabilitering, stöd för implementering av åtgärdskoder och förslag till åtgärder [Continued development of cancer rehabilitation: Survey of cancer rehabilitation in the regions, support for the implementation of intervention codes and suggested action]. Stockholm: Socialstyrelsen; 2024.

[40] Johansson B, Berglund G, Hoffman K, et al. The role of the general practitioner in cancer care and the effect of an extended information routine. Scand J Prim Health Care. 2000;18:143–8. 10.1080/028134300453331. 11097098

[41] Garpenhag L, Halling A, Calling S, et al. Being ill was the easy part: Exploring cancer survivors’ reactions to perceived challenges in engaging with primary healthcare. Int J Qual Stud Health Well-being. 2024;19(1):2361492. 10.1080/17482631.2024.2361492. PMC11146241 38824662

[42] Vård- och omsorgsanalys. Kontinuitet och fast läkarkontakt: Kartläggning av måluppfyllelsen i överenskommelserna om en god och nära vård. [Continuity and regular physicians: A survey of goal achievement in the agreements about high quality and proximate care]. Stockholm: Myndigheten för vård- och omsorgsanalys. Swedish; 2023.

[43] Harris PA, Taylor R, Thielke R, et al. Research electronic data capture (REDCap) – A metadata-driven methodology and workflow process for providing translational research informatics support. J Biomed Inf. 2009;42(2):377–81. 10.1016/j.jbi.2008.08.010. PMC2700030 18929686

[44] Harris PA, Taylor R, Minor BL, et al. The REDCap consortium: Building an international community of software partners. J Biomed Inf. 2019;95:103208. 10.1016/j.jbi.2019.103208. PMC7254481 31078660

[45] Aaronson NK, Ahmedzai S, Bergman B, et al. The European Organisation for Research and Treatment of Cancer QLQ-C30: A quality-of-life instrument for use in international clinical trials in oncology. J Natl Cancer Inst. 1993;85:365–76. 10.1093/jnci/85.5.365 8433390

[46] Ohlsson-Nevo E, Fogelkvist M, Lundqvist LO, et al. Validation of the Assessment of Rehabilitation Needs Checklist in a Swedish cancer population. J Patient Rep Outcomes. 2024;8(1):142. 10.1186/s41687-024-00818-5. PMC11621288 39638928

[47] Ware JE Jr, Sherbourne CD. The MOS 36-Item Short-Form Health Survey (SF-36): I. Conceptual Framework and Item Selection. Med Care. 1992;30:473–83. 1593914

[48] Fayers PM, Aaronson NK, Bjordal K et al. The EORTC QLQ-C30 Scoring Manual (3rd Edition). Brussels: European Organisation for Research and Treatment of Cancer; 2001.

[49] Hilbe JM. Practical Guide to Logistic Regression. Boca Raton, Fl: CRC; 2015.

[50] Vård- och omsorgsanalys. Vården ur befolkningens perspektiv: International Health Policy Survey (IHP) 2023. [The healthcare system from the perspective of the population: International Health Policy Survey (IHP) 2023]. Stockholm: Myndigheten för vård- och omsorgsanalys. Swedish; 2024.

[51] Vård- och omsorgsanalys. Med örat mot marken: Förslag på nationell uppföljning av hälso- och sjukvården Del 2: Resultatredovisning [With the ear to the ground: Proposal for a national evaluation of health care, part 2: Results]. Stockholm: Myndigheten för vård- och omsorgsanalys. Swedish; 2019.

[52] Ritchie CS, Kvale E, Fisch MJ, Multimorbidity. An issue of growing importance for oncologists. J Oncol Pract. 2011;7(6):371–4. 10.1200/JOP.2011.000460. PMC3219463 22379419

[53] Jaipaul CK, Rosenthal GE. Are older patients more satisfied with hospital care than younger patients? J Gen Intern Med. 2003;18(1):23–30. 10.1046/j.1525-1497.2003.20114.x. PMC1494807 12534760

[54] Collaço N, Lippiett KA, Wright D, et al. Barriers and facilitators to integrated cancer care between primary and secondary care: A scoping review. Support Care Cancer. 2024;32:120. 10.1007/s00520-023-08278-1. PMC10803398 38252169

[55] Saultz JW, Albedaiwi W. Interpersonal continuity of care and patient satisfaction: a critical review. Ann Fam Med. 2004;2(5):445–51. 10.1370/afm.91. PMC1466724 15506579

[56] Lautamatti E, Sumanen M, Raivio R, et al. Continuity of care is associated with satisfaction with local health care services. BMC Fam Pract. 2020;21(1):181. 10.1186/s12875-020-01251-5. PMC7487808 32887566

[57] Chu AK, Mutsaers B, Lebel S. The association between survivorship care plans and patient-reported satisfaction and confidence with follow-up cancer care provided by primary care providers. Curr Oncol. 2022;29:7343–54. 10.3390/curroncol29100577. PMC9600139 36290854

[58] Perneger TV, Peytremann-Bridevaux I, Combescure C. Patient satisfaction and survey response in 717 hospital surveys in Switzerland: a cross-sectional study. BMC Health Serv Res. 2020;20(1):158. 10.1186/s12913-020-5012-2. PMC7052977 32122346

